# Foot loading of an African population

**DOI:** 10.1186/1757-1146-5-S1-O25

**Published:** 2012-04-10

**Authors:** Niki M Stolwijk, Jacques Duysens, Jan-Willem K Louwerens, Noel LW Keijsers

**Affiliations:** 1 Department of RD&E, Sint Maartenskliniek, Nijmegen, the Netherlands; 2 Orthopaedics, Sint Maartenskliniek, Nijmegen, the Netherlands; 3 Research Center for Movement Control and Neuroplasticity, Department of Biomedical Kinesiology, Katholieke Universiteit Leuven, Leuven, Belgium

## Background

In contrast to western countries, foot complaints are rare in Africa. This is remarkable, as most Africans load their feet significantly more; they walk many hours each day, often barefoot or with worn-out shoes. The reason why Africans can withstand such loading without developing foot complaints might be related to the way the foot is loaded. Therefore, foot shape and dynamic plantar pressure distribution of an African population was compared to a Caucasian population.

## Materials and methods

The plantar pressure distribution of 77 persons from Malawi (Blantyre and surroundings) and 77 Dutch persons were measured using a USB (in Malawi) and 3D (in the Netherlands) Foot Scan® pressure plate (Rsscan Int.). None of the subjects reported foot complaints. The normalized [1] mean pressure (MP), peak pressure (PP) and pressure-time integral (PTI) as well as the Arch Index (AI) and the trajectory of the centre of pressure (COP) during the stance phase were calculated and compared between both groups. Standardized pictures were taken from the feet to assess the medial arch angle.

## Results

The MP, PP and PTI were significantly higher under the midfoot and lower under the heel and metatarsal head II and III for the Malawian group (p<0.007). Furthermore the AI was significantly higher in the Malawian group (mean 0.28 (SD 0.03) compared to the Dutch group (mean 0.21 (SD 0.06). The COP trajectory was situated more anteriorly during the first and last part of stance and more posteriorly during the middle part of the stance phase. In the Malawi group, the medial arch angle was significantly larger (p<0.05).

## Conclusions

Africans have a different loading pattern compared to Caucasians, with less loading on the forefoot and heel and more contribution of the midfoot and toes during the roll off. This loading pattern generates a more equal distribution of pressure, which might help to prevent for foot complaints.
